# Comparison of Early Feeding Practices in Mother–Father Dyads and Possible Generalization of an Efficacious Maternal Intervention to Fathers’ Feeding Practices: A Secondary Analysis

**DOI:** 10.3390/ijerph17176075

**Published:** 2020-08-20

**Authors:** Lynne A. Daniels, Kimberley M. Mallan, Elena Jansen, Jan M. Nicholson, Anthea M. Magarey, Karen Thorpe

**Affiliations:** 1 School of Exercise and Nutrition Sciences, Queensland University of Technology, Brisbane, QLD 4059, Australia; Kimberley.Mallan@acu.edu.au; 2 Institute of Health and Biomedical Innovation, Queensland University of Technology, Brisbane, QLD 4059, Australia; 3 School of Behavioural and Health Sciences, Australian Catholic University, Brisbane, QLD, 4014, Australia; 4 School of Medicine, Johns Hopkins University, Baltimore, MD 21287, USA; 5 Judith Lumley Centre, College of Science, Health and Engineering, La Trobe University, Melbourne, VIC 3086, Australia; J.Nicholson@latrobe.edu.au; 6 Nutrition and Dietetics, School of Health Sciences, Flinders University, Adelaide, SA 5001, Australia; antheamagarey@gmail.com; 7 School of Psychology and Counselling, Queensland University of Technology, Brisbane, QLD 4059, Australia; k.thorpe@uq.edu.au; 8 Institute for Social Science Research, The University of Queensland, Brisbane, QLD 4068, Australia

**Keywords:** fathers, mothers, child feeding practices, intervention

## Abstract

To compare feeding practices within mother–father dyads and explore whether outcomes of an efficacious intervention for mothers generalised to fathers’ feeding practices. The NOURISH RCT evaluated an early feeding intervention that promoted positive feeding practices to support development of healthy eating habits and growth. The intervention was delivered to first-time mothers via 2 × 12 week modules commencing when children were 4 and 14 months. Mothers self-reported feeding practice outcomes at child age 2 years using validated scales (1 = low to 5 = high) from the Child Feeding Questionnaire (CFQ). Nine months later, an independent cross-sectional descriptive study to investigate fathers’ feeding practices was initiated. Fathers were recruited by contacting (via letter) mothers participating in two pre-existing studies, including the NOURISH trial. Fathers completed a feeding practices questionnaire, similar to that used for NOURISH outcome assessments. Seventy-five fathers recruited via the NOURISH cohort (21% response) returned questionnaires. Response data from this subset of fathers were then linked to the corresponding NOURISH maternal data. Complete data were available from 70 dyads. Compared with mothers, fathers self-reported higher concern about child overweight (2.2 vs. 1.3), restriction (3.6 vs. 2.9) and pressure (2.6 vs. 2.1), all *p* < 0.001. Fathers whose partners were allocated to the intervention group used less pressure (mean difference 0.46, *p* = 0.045) and were more willing to let the child decide how much to eat (−0.51, *p* = 0.032). Fathers’ higher concern about child weight and more frequent use of non-responsive feeding practices, when compared with mothers, identify them as potentially potent contributors to child feeding. This preliminary evidence for modest generalisation of an efficacious maternal intervention to apparent effects on some paternal feeding practices speaks to the importance and promise of including fathers in early feeding interventions.

## 1. Introduction

The need to prevent childhood obesity is widely accepted. There is a strong rationale and growing body of evidence suggesting that infancy, when biology and behaviour are plastic, provides a critical ‘sensitive’ period in which to intervene [1,2,3]. Early child feeding practices are associated with child obesity risk [4] and are potentially modifiable [1,5]. In conjunction with genetic factors [3], children’s early feeding experience—the ‘what’ and ‘how’ of feeding—shapes children’s food preferences, eating behaviours, and dietary intake and lays the foundation for lifelong eating patterns [4,6,7,8]. While infants have an inborn capacity to self-regulate their energy intake to need [9], early feeding practices that are non-responsive to the child’s cues of hunger and satiety can undermine this intrinsic self-regulation. Such non-responsive feeding practices, also widely referred to as ‘controlling’, include pressure, reward, restriction and/or emotional feeding (using food to calm) and teach children to eat for reasons unrelated to appetite. Conversely, responsive feeding practices are postulated to support and foster children’s ability to respond appropriately to internal cues of hunger and satiety, conserving their ability to self-regulate [1,9,10,11].

To date, research into childhood obesity treatment and prevention, including the role of parental feeding practices, has largely focused on mothers [12,13,14]. With changes to the gender-based divisions of responsibilities for employment and parenting within families and society at large, fathers have an increasingly important role in early feeding. However, they are largely absent from both descriptive [12] and intervention studies [13] that aim to understand parental feeding practices and prevent childhood obesity. A review by Khandpur et al. [12] identified 20 reports (all descriptive, two qualitative) providing independent data on fathers’ feeding practices. Fourteen of these were in pre-schoolers, but only two studies had sample sizes >200 [15,16]. Five of the eight studies that compared feeding practices of parents with pre-schoolers reported differences, including fathers reporting lower responsibility and greater concern, restriction and pressure [12].

A recent review identified and evaluated 213 interventions designed to prevent (51%) or treat obesity in 0–18 year olds [13]. In the 80 studies where participation was limited to one parent per family, only 6% of participants were fathers. Of the 133 studies where the intervention was open to one/both parents, only 8 (11%) studies reported quantitative baseline data on father and none reported attendance of fathers. The 12 studies in infants (0–12 months) recruited mothers only and just 10% of participants with 2–4-year-old children were fathers (*n* = 26 studies). The only RCT that specifically targeted fathers evaluated a treatment program for 93 fathers with overweight/obesity and their 5–12-year old children of any weight status (*n* = 139) and showed modest positive intervention effects on adiposity of both fathers and children [17].

Despite the resounding absence of fathers from research related to child feeding practices and obesity risk, there is growing evidence that the contribution of fathers to the early feeding environment and child obesity risk are important. Fathers contribute to family food selection. For instance, fathers’ dietary quality (e.g., fruit and vegetable intake) has been associated with child diet [18,19]. In a large, nationally representative sample (*N* = 4983), parenting behaviours and styles of fathers, but not mothers, were associated with higher child weight status [20]. Furthermore, fathers’ weight status appears to be differentially associated with child obesity risk. In a large study (*N* = 3285), relative to children (8–9 years old) with both parents of healthy weight, children of a father with overweight or obesity and a mother of healthy weight were at much greater risk of obesity (OR 4 and OR 15 respectively), whilst the reverse (mother with overweight/obesity and father of healthy weight) was not associated with child obesity risk [21]. Failure to include fathers in interventions potentially represents more than just a gap in the evidence. Such exclusion may result in unintended consequences such as attenuation of intervention effects if fathers directly or indirectly undermine mothers’ capacity to implement intervention strategies. Targeting mothers but not fathers may exacerbate gender-based food labour imbalances [22,23], parenting stereotypes, discordant parenting and family conflict. Furthermore, it misses an opportunity to enhance positive engagement of men in their own and their families’ health and wellbeing.

Given the complete absence of fathers from early feeding interventions to date [17], the potential responsiveness of fathers to interventions and how this compares to maternal outcomes is unknown. This lack of evidence presents challenges in designing and acquiring funding for RCTs that recruit and deliver acceptable and potentially effective interventions to both parents. One preliminary step in building evidence to support the additional effort and cost of including fathers in interventions is to explore whether inventions targeting the feeding practices of mothers can generalise to fathers’ attitudes and practices. Such evidence would suggest that fathers’ feeding practices are potentially responsive to intervention. The NOURISH RCT allocated 698 first-time mothers with healthy term infants and evaluated an early feeding intervention that promoted positive feeding practices in the context of developing healthy eating habits and obesity prevention. The intervention was delivered from infant age 4–16 months. Follow up when the children were 2, 3.7 and 5 years old demonstrated a strong, consistent and sustained positive intervention effect on maternal feeding practices [1,5,24]. The secondary analysis presented here utilises data from a subset of NOURISH mother-child dyads, and fathers of the study child recruited for a subsequent independent study. The aims of the current study were to compare feeding practices within mother–father dyads and explore whether the positive intervention impact on maternal feeding practices generalised to any indirect effects on fathers’ feeding practices.

## 2. Materials and Methods

### 2.1. Study Design and Participants

This study is a secondary analysis of data from parent dyads, the mothers of whom were a subsample of participants in the NOURISH RCT [1,5], described above. NOURISH participants (*N* = 698) were recruited on postnatal wards in two Australian cities (Brisbane and Adelaide), via consecutive sampling. Baseline assessment and independent random allocation occurred, and intervention commenced when their infants were aged approximately 4 months. Mothers self-reported feeding practices at infant ages 14 months (mid-intervention), 24 months (6 months post intervention completion), 3.7 and 5 years of age. Despite evidence of some selection bias with respect to age, education and marital status, there were no baseline differences in characteristics of mothers or children by group allocation [1,5].

The NOURISH trial primarily targeted mothers and although fathers were invited to engage with the intervention, very few did so. To better understand this issue, approximately 3 years after NOURISH commenced and 9 months post the RCT 24-month-old outcome assessment, we undertook an independent cross-sectional descriptive study—‘Fathers’ Feeding Practices and Participation (FFPP) Study’—which aimed to *“investigate fathers’ attitudes to and involvement in feeding their young children”* [15,25]. Fathers were recruited by contacting mothers participating in two pre-existing studies (including NOURISH) and via convenience sampling of university staff and students [15,25]. All NOURISH mothers living in Brisbane (*N* = 362) from both the control and intervention groups were sent a letter of invitation and questionnaire to pass on to the “father or male caregiver” of the trial child. Seventy-five fathers recruited via the NOURISH cohort (21% response rate) returned a questionnaire. We then linked common response data from this subset of fathers to the corresponding maternal responses collected at the 24-month-old NOURISH RCT outcome assessment. Due to missing data (on feeding practices), complete data for 70 mother–father dyads were available for analysis (control: *n* = 37, intervention: *n* = 33).

### 2.2. NOURISH Intervention

The intervention [1,26] was delivered to mothers via two modules of six group sessions over twelve weeks commencing when the infants were 4 and 14 months old. The group sessions were co-led by study dietitians and psychologists who received standardised training and used comprehensive, standardised facilitator and participant materials. The intervention promoted positive feeding practices in the context of obesity prevention and promoted (i) neutral increased exposure to a wide range of healthy foods and limited exposure to unhealthy foods to foster healthy food preferences, (ii) responsive feeding that encouraged feeding to infant cues of hunger and satiety to teach infants to eat in response to appetite and support their intrinsic regulation of intake, and (iii) authoritative/responsive parenting [11]. Messages to participants focused on healthy growth and eating patterns rather than obesity prevention *per se* [26]. Participants allocated to the control group had self-directed access to standard community care that, at mothers’ initiative, potentially included child weighing and telephone or internet advice.

### 2.3. Measures

#### 2.3.1. Child Feeding Attitudes and Practices

For both mothers and fathers, feeding attitudes and use of (non-)responsive feeding practices were independently self-reported via four scales (1 = lowest to 5 = highest) from the widely-used Child Feeding Questionnaire (CFQ) [27]: *perceived responsibility* (3 items) assessed parents’ attitudes towards their role in feeding their child, *concern about child weight* (3 items) assessed attitudes toward their child’s weight status and eating behaviour in the context of obesity proneness, *pressure to eat* (4 items) and *restriction* (8 items) assessed controlling feeding practices. Both parents completed two additional items, used previously [1,5], that assessed Satter’s [28] conceptualisation of responsive feeding (‘parent provides, child decides’)—(i) *Who decides what your child eats—you or your child?*, and (ii) *Who decides how much food your child eats—you or your child*? (1 = you only to 5 = your child only). Higher scores indicated greater child control in terms of what and how much the child eats. Mean scores for each scale/item were used.

#### 2.3.2. Participant Characteristics

Fathers’ self-reported characteristics included education, their own and their NOURISH child’s age, and their own height and weight. Fathers’ age at the birth of the child was estimated by subtracting child age (months) from their self-reported age (years). Maternal and infant characteristics, collected at the first postnatal contact, included maternal age at delivery, education and infant birth weight (from hospital records). At follow-up assessments, maternal and child weights and heights (child standing) were measured by trained study staff using standard procedures. Infant birth weight and Body Mass Index (BMI) for age z-scores were calculated using the World Health Organization Anthro software program version 3.0.1 and macros [29].

All subjects gave their informed consent for inclusion before they participated in the study. The NOURISH RCT and Fathers’ Feeding Practices and Participation Study were approved by Queensland University of Technology Human Research Ethics Committee (NOURISH: HREC 00171 Protocol 0700000752, approved on 28 September 2007). The NOURISH RCT was also approved by the relevant ethics committees at all the recruitment hospitals and Flinders University and registered in the Australian New Zealand Clinical Trials Registry (ACTRN 12608000056392).

### 2.4. Statistical Analysis

Demographic characteristics of the intervention and control group fathers and mothers were compared using independent samples *t* tests (continuous variables) and likelihood ratio chi-square tests (dichotomous variables) (Table 1). As fathers’ and mothers’ feeding practices were reported in relation to the same child, these were compared using paired samples *t*-tests (Table 2). All between-gender (fathers vs. mothers) comparisons were stratified by NOURISH group (intervention vs. control) to establish whether any detected parent differences showed a similar pattern across both conditions. Within-gender differences (fathers, mothers) in the feeding practices by intervention and control group were compared using independent samples *t*-tests. No adjustment for covariates was made due to absence of significant differences between the intervention and control groups on these variables (Table 1). All analyses were conducted in IBM SPSS Statistics (version 24) and a significance level of *p* < 0.05 (two-tailed) was used throughout.

## 3. Results

Characteristics of fathers, mothers and children (*N* = 70) in this study are presented in Table 1. All fathers and mothers were the biological parent of the NOURISH child. Children (43% male) were on average 24 months old (*SD* = 1) when mothers completed the feeding practices questionnaire as part of the NOURISH trial, and 33 months old (*SD* = 5) when fathers completed the corresponding questionnaire. On average, fathers were 34 (*SD* = 5) and mothers were 32 (*SD* = 5) years old at the child’s birth. Forty-five percent of fathers and 76% of mothers had completed a university degree. Self-reported mean BMI for fathers was 26 (*SD* = 4) and measured mean BMI at baseline (4 months postpartum) for mothers was 25 (*SD* = 4). There were no differences between control and intervention fathers and mothers on these key characteristics, all *p*-values >0.05.

Comparison of mothers in this sub-study with the full NOURISH sample revealed several differences on maternal characteristics. Compared to the full sample, mothers in the present sample were on average two years older at delivery (NOURISH full sample: 30 years, *SD* = 5), a higher proportion were university educated (NOURISH full sample: 58%) and they had a lower mean BMI at baseline (NOURISH full sample: 26, *SD* = 5).

Comparisons between fathers’ and mothers’ feeding attitudes and practices are presented in Table 2 for control and intervention groups separately. For the between-gender comparisons (fathers vs. mothers), the pattern of findings did not differ according to intervention group allocation. Overall, fathers’ perceived responsibility for child feeding was significantly lower (*M* = 2.41, *SD* = 0.90) than mothers’ (*M* = 4.19, *SD* = 0.57), yet fathers were more concerned than mothers about the child becoming overweight (*M* = 2.18, *SD* = 0.93 vs. *M* = 1.28, *SD* = 0.65), and used more restriction (*M* = 3.62, *SD* = 0.53 vs. *M* = 2.93, *SD* = 0.71) and more pressure (*M* = 2.61, *SD* = 0.96 vs. *M* = 2.09, *SD* = 0.94), all *p*-values <0.001. There were no differences between parents in terms of the degree to which child vs. parent made decisions about what the child eats (fathers: *M* = 2.19, *SD* = 0.73, mothers: *M* = 2.29, *SD* = 0.66, *p* = 0.30), but fathers rated themselves as less willing to let the child decide how much to eat than mothers (fathers: *M* = 2.91, *SD* = 0.97, mothers: *M* = 3.73, *SD* = 0.96, *p* < 0.001).

Consistent with findings from the full NOURISH sample [5], mothers in this sub-study showed significant differences in self-reported feeding practices by intervention condition. Mothers in the intervention group were less concerned about the child becoming overweight (mean difference = 0.33, 95%*CI* = −0.22 to 0.33, *p* = 0.027) and more willing to let the child decide how much to eat (mean difference = −1.14, 95%*CI* = −1.51 to −0.78, *p* < 0.001) than control group mothers. There was also a non-significant trend for intervention group mothers to use less pressure (mean difference = 0.32, 95%*CI* = −0.13 to 0.76, *p* = 0.16). Mothers did not vary on the other measures (*p*-values ≥ 0.32). Fathers whose partners were in the intervention group reported using significantly less pressure (mean difference = 0.46, 95%*CI* = 0.01 to 0.91, *p* = 0.045), and were more willing to let the child decide how much to eat (mean difference = −0.51, 95%*CI* = −0.96 to −0.05, *p* = 0.032). No other differences for fathers were significant (*p*-values ≥ 0.25).

## 4. Discussion

This secondary analysis sought to provide much needed data on fathers’ feeding practices by recruiting fathers indirectly via participants in a pre-existing efficacious intervention study that targeted mothers. The study firstly compared feeding practices across mother–father dyads when their child was 2–3 years old. It then sought to examine differences in practices between fathers in intervention and control families to assess the potential generalisation of positive maternal intervention outcomes to fathers’ feeding practices.

Mothers reported greater perceived responsibility for child feeding than fathers, consistent with both gender-role stereotypes and empirical evidence regarding mothers’ and fathers’ roles in child feeding [12,31,32]. In contrast, fathers were more concerned about their child becoming overweight and correspondingly reported more controlling feeding practices than mothers in terms of pressure, restriction, and deciding how much their child should eat. Fathers whose partners were in the intervention arm of the NOURISH RCT [1,5] self-reported exerting less pressure and control over how much their children eat than fathers whose family had no exposure to the intervention.

Overall we found that, compared to mothers, fathers self-reported more controlling feeding practices that have been associated with increased obesity risk [4,9,10,12] and, hence, represent comparatively suboptimal paternal feeding practices. Other comparable studies in preschool children are variable in terms of tools and constructs considered as well as findings. Four studies [31,33,34,35] from the same research group in England, report data on the CFQ scales [27] for pressure and restriction only. Sample sizes range from 23–107 parent dyads with mean child age 37–42 months, but the extent of participant sample overlap across the reports is unclear. All studies had a focus on associations amongst feeding practices and mental health or eating behaviour symptomatology. None report any parental differences in pressure and restriction. The only study [31] to report the CFQ responsibility scale [27] shows that mothers (*N* = 94 dyads) clearly perceived higher responsibility for child feeding than fathers (4.1 vs. 2.5), similar to our results (mothers 4.2 vs. 2.4). One study [36] that aimed to examine links between parent ‘anti-fat’ attitudes and restrictive feeding in 4–6 year-olds, reported that, despite there being no difference in parent (*N* = 102 dyads) concern about child weight (using the CFQ [27]), fathers scored higher on a ‘restriction for weight’ subscale. Hendy et al. [16] in a US study designed to develop and validate a new feeding practices assessment tool, compared feeding practices within 272 parent dyads with children 4.5 years of age. Fathers more often used ‘insistence on eating’ while mothers more often used ‘positive persuasion’ to eat and a range of strategies to enhance dietary quality (e.g., ‘snack limits’). Two recent Australian studies [37,38] that utilised the Feeding Practices and Structure Questionnaire (FPSQ) [39,40], found no mother–father (*n* = 279 mothers, *n* = 225 fathers [37]; *N* = 208 parent dyads [38]) differences in three non-responsive feeding practices subscales (persuasive feeding, reward for eating, overt restriction). However, in the study that compared practices within parent dyads [38] fathers reported more frequently using reward for behaviour. In summary, the amount and quality of evidence regarding the scope and magnitude of disparities in feeding practices between parents is limited and the findings mixed.

Encouragingly, fathers whose partners received the intervention reported using less pressure and more child control of amount eaten than those whose partners were allocated to the control group. These apparent intervention effects are consistent with those on maternal feeding practices in the full NOURISH outcome analysis at 2 years of age [5]. Intervention mothers were provided with purpose-designed written summaries of key intervention parenting and feeding messages/strategies and explicitly encouraged to discuss these with other carers, particularly fathers. As such, generalisation of intervention effect is a plausible explanation. However, caution is required as we do not have baseline data on fathers and cannot rule out the influence of other pre-existing biases. Nevertheless, these data are promising and provide the first preliminary evidence that paternal feeding practices may be modifiable, even via indirect exposure to an intervention.

This study has several limitations. It is a secondary analysis of a convenience sub-sample drawn from two pre-existing studies. It is possible the 9-month gap in child age between collection of maternal (24 months) and paternal (33 months) reports of child feeding practices could explain the differences observed between mothers and fathers. Longitudinal analysis of data from the NOURISH trial [1,41] indicates that maternal use of controlling feeding practices, including three of the four CFQ scales used here (perceived responsibility, pressure and restriction), increased with age, particularly from 2–3.7 years of age. However, the magnitude of these changes in maternal scale scores over 20 months (child age 24 to 44 months) are around 0.2–0.3 in both the control and intervention groups [1]. Given the magnitude of the differences in the mean scores reported by parents 9 months apart are 2–6-fold greater (0.5–1.8) than these age-related increases in maternal scores, it seems unlikely that the differences in parent feeding practices can be attributed to the time lag in reporting of fathers’ feeding practices alone. Nevertheless, we cannot rule out this possibility.

Further limitations include the small sample size and the low participation rate (21%) of fathers. Cross-sectional descriptive studies, such as the analyses presented here, commonly rely on convenience sampling via web-based platforms or distribution of written information via childcare centres, kindergartens and the like. As such estimations of response rates and selection bias are frequently not reported. Of the comparable studies discussed above four [17,33,34,38] did not report a response rate and the rates for the remaining studies [16,31,35,36] ranged from 9–35%. For six of eight of these studies [17,31,33,34,35,36] the number of dyads ranged from 23–107, and the remaining two [16,38] had 272 and 208 (consent rate 15% and not reported) respectively. As such our sample size (70 dyads) and consent rate (21%) are within the scope of other directly relevant studies. Nevertheless, it is possible that participating parents were a non-representative ‘concerned’ group. Comparison of the mothers included in this sub-study with the larger NOURISH sample [5], indicated selection bias in terms of age, education and BMI. Compared to the larger fathers study [15], fathers here were of similar age but more likely to have a university degree (45% vs. 34%) The use of self-report measures coupled with the volunteer/selection bias may have resulted in more socially desirable responses from participants. The extent to which theses biases differentially effect maternal versus paternal responses and hence the differences in practices described here is unknown. Although fathers were asked to complete the questionnaire independently, we cannot be sure that this was always the case. However, maternal influence on fathers reporting would tend to attenuate differences. There were no control vs. intervention differences on key characteristics within the sub-sample of mothers and fathers whose responses are analysed here. Hence, the intervention effects are likely to have internal validity. Nevertheless, this subsample is not representative of the total sample from either of the source studies and overall the generalisability of the present results beyond parents of toddlers from relatively highly educated backgrounds in Australia is unclear. The study’s strengths include examination of six different feeding practices compared to 2–3 considered in other studies. It is the first study to examine the potential for indirect generalisation to fathers’ feeding practices of the effects of an early feeding intervention delivered to mothers.

Given what is known regarding the potentially detrimental impacts of controlling feeding practices on children’s eating and weight [4,9,10,12], our findings raise several issues regarding current approaches to early feeding interventions. First, the efficacy of child obesity prevention interventions that successfully modify mothers’ feeding practices [5,12] may be attenuated if the feeding practices of fathers are not also targeted, particularly given our own and others’ evidence [12,38] that fathers comparatively use more non-responsive controlling practices. Intervening with mothers appeared to generalise to a modest indirect effect on two of the six feeding practices of fathers that we assessed. Nevertheless, these results are encouraging, and it is plausible that more substantial change could be achieved through the direct engagement of fathers in child feeding interventions.

Whilst that rationale to include fathers in early feeding and obesity prevention interventions is strong, there are numerous challenges. Effective engagement of fathers will likely require more than simply offering access to interventions designed for mothers. There is a need to identify, develop and evaluate (process and impact) effective strategies that target fathers including (i) recruitment methods, (ii) feasible delivery modes and settings, (iii) intervention messages that are relevant and acceptable and (iv) outcome measures and methods validated for use in fathers. Intervention messages and focus may need to vary between parents to targets strengths and interests [17]. However, it is critical that intervention approaches do not inadvertently contribute to discordant parenting or parental conflict and that within the intervention context parents are supported to work together to achieve positive intervention outcomes. Cultural and ethnic background, a range of sociodemographic factors, and historical feeding interactions [42] are likely to influence the roles of mothers and fathers in child rearing and feeding. Hence, ensuring diversity in parents participating in interventions will be critical to building a robust evidence base for the role of early feeding practices of both parents. More research is required to understand fathers’ enrolment and retention patterns, potential outcome effect sizes and interactions with maternal responses to support sample size calculations, comprehensive and robust evaluation plans and high-quality competitive grant applications.

## 5. Conclusions

The outcomes of this study suggest that fathers, compared to mothers, reported lower perceived responsibility for child feeding and, despite reporting more concern about child weight status, paradoxically appear to more frequently use more controlling and, hence, suboptimal feeding practices. As such, they are potentially a potent influence on child feeding. This study found preliminary evidence that suggests generalisation of an efficacious maternal early feeding intervention to modest indirect positive effects on fathers’ feeding practices. The low participation rate of fathers, possible selection of a ‘concerned’ sample and the 9-month gap in child age between maternal and paternal data collection indicate caution in extrapolation of these results beyond the small study sample. Nevertheless, these preliminary results are encouraging, and suggest there is a need to and potential benefits from directly engaging both parents in child obesity prevention and treatment interventions [12,21]. Effectively recruiting and engaging fathers in early feeding interventions will be potentially challenging [43]. It will require identification and evaluation of recruitment strategies, acceptable intervention mode, messages and settings and validated outcome tools and methods for use in fathers. In terms of early feeding interventions, fathers are the ‘forgotten’ parent and this study suggests it is necessary and potentially useful to bring them to the intervention table.

## Figures and Tables

**Table 1:** Characteristics of study participants (*N* = 70 parent dyads).

Characteristics	NOURISH RCT Allocation ^1^	
	Control	Intervention
	(*n* = 37 parent dyads)	(*n* = 33 parent dyads)
	*M* (*SD*) or %	
*Mother*		
Age at delivery (years)	32 (6)	32 (4)
Education (University)	68	85
BMI ^2^	25 (5)	24 (3)
*Father*		
Age at child’s birth (years)	34 (5)	34 (5)
Education (University) *n* = 69	50	39
BMI (self-reported) *n* = 69	26 (4)	25 (3)
*Child*		
Birth weight Z-score	0.28 (0.70)	0.41 (0.77)
Gender (male)	46	40
BMI Z-score at 2 years	0.85 (0.95)	0.55 (0.92)

Abbreviations: *M* = mean, *SD* = standard deviation; *n* = sample size due to missing data; ^1^ the NOURISH RCT evaluated an early feeding intervention for first-time mothers, mother-child dyads were allocated [30]; ^2^ measured BMI at NOURISH baseline assessment approximately 4 months postpartum.

**Table 2:** Fathers’ versus mothers’ (*N* = 70 dyads) self-reported feeding attitudes and practices^1,2^ (*M*, *SD*) stratified by NOURISH group allocation (control vs. intervention) [1,5].

Child Feeding Attitudes/Practices	Father	Mother	Mean Difference	*p*-Value *
	*M (SD)*		(95% *CI*)	
Control (*n* = 37 dyads)				
Perceived responsibility ^1^	2.47 (0.94)	4.22 (0.58)	−1.75 (−2.19, −1.30)	<0.001
Concern about child becoming overweight ^1^	2.30 (1.03)	1.43 (0.83) ^a^	0.86 (0.50, 1.23)	<0.001
Restriction ^1^	3.60 (0.49)	3.01 (0.83)	0.59 (0.24, 0.93)	0.001
Pressure to eat ^1^	2.82 (0.87) ^b^	2.24 (0.88)	0.58 (0.21, 0.95)	0.003
Who decides what child eats ^2^	2.22 (0.58)	2.24 (0.64)	−0.03 (−0.24, 0.19)	0.800
Who decides how much child eats ^2^	2.68 (0.82) ^c^	3.19 (0.91) ^d^	−0.51 (−0.87, −0.16)	0.006
Intervention (*n* = 33 dyads)				
Perceived responsibility ^1^	2.34 (0.86)	4.16 (0.56)	−1.82 (−2.25, −1.38)	<0.001
Concern about child becoming overweight ^1^	2.04 (0.81)	1.10 (0.27) ^a^	0.94 (0.64, 1.24)	<0.001
Restriction ^1^	3.63 (0.57)	2.85 (0.55)	0.79 (0.52, 1.05)	<0.001
Pressure to eat ^1^	2.36 (1.02) ^b^	1.92 (0.99)	0.44 (0.05, 0.83)	0.03
Who decides what child eats ^2^	2.15 (0.87)	2.33 (0.69)	−0.18 (−0.52, 0.16)	0.28
Who decides how much child eats ^2^	3.18 (1.07) ^c^	4.33 (0.60) ^d^	−1.15 (−1.50, −0.81)	<0.001

*M* = mean, *SD* = standard deviation; * based on paired samples *t*-tests; ^a,b,c,d^ independent *t*-tests to determine allocation group effects (control vs. intervention) within maternal and paternal responses; same letter indicates *p* < 0.05. See next section of manuscript for further details; ^1^ Child Feeding Questionnaire [27]; response options: 1 (lowest) to 5 (highest); ^2^ items based on Satter’s [28] Division of Responsibility in Feeding Model (i.e., parent provides, child decides); response options 1 (parent always decides) to 5 (child always decides) [1,5].
